# The psychosocial matters: a call for attachment theory in kidney health research

**DOI:** 10.1080/0886022X.2026.2666442

**Published:** 2026-05-13

**Authors:** Devron J. Swaby, Ana Samudio, Robert Maunder, Jon Hunter, Istvan Mucsi

**Affiliations:** a Ajmera Transplant Centre, University Health Network, Toronto, Canada; b School of Interdisciplinary Science, McMaster University, Hamilton, Canada; c Department of Psychiatry, Sinai Health System, Toronto, Canada; d Department of Medicine, University of Toronto, Toronto, Canada; e Pencer Chair in Applied General Psychiatry, University of Toronto, Toronto, Canada; f Division of Nephrology, University Health Network, Toronto, Canada

**Keywords:** Attachment theory, chronic kidney disease, biopsychosocial, patient-centered care, patient-reported outcomes

## Introduction

With increasing global incidence, prevalence, and mortality, chronic kidney disease (CKD) presents an international public health concern, affecting over 14% of the global adult population, particularly those in low and middle-income countries [1,2]. Patients with CKD, especially those with advanced CKD without kidney transplant (KT), experience significant mental and physical symptoms, including depression, anxiety, fatigue, sleep problems, chronic pain, and impaired physical functioning, which are associated with low health-related quality of life (HRQoL) [3]. Patient-reported outcomes like HRQoL are important factors for patients with CKD, and there is a need for patient-centered, whole-person approaches in kidney care [4–6].

Adult attachment is associated with mental health, health behaviors, and patient–clinician relationships among patients with chronic diseases [7]. Attachment theory has been proposed as a unifying framework for understanding the psychosomatic links between stress, illness behavior, and chronic disease [8]. Early interactions with caregivers shape relatively stable attachment patterns, reflecting expectations of self and others, which are present across interactions but become particularly salient in moments of perceived threat or vulnerability, such as in the context of health and disease. In these contexts, an individual’s unique attachment patterns may influence their stress response, coping behaviors, use of support, and treatment adherence [9]. In turn, these processes may contribute to the presentation and course of their disease, while the illness context itself may further reinforce attachment-related responses. This bidirectional relationship between attachment and physical illness trajectory has been detailed in prior work [7–11]. As a component of a biopsychosocial framework, attachment theory may therefore help us to better understand patients’ health needs and advance patient-centered kidney care.

## Attachment theory

Attachment theory [12–15] provides a lens to examine how variations in individuals’ emotional, relational, and social functioning play a role not only in one’s intimate relationships but also in health and disease [8,9,11]. Attachment is an evolutionarily based system designed to ensure that infants maintain proximity to caregivers during times of threat [12,15]. When caregiving needs are met, and infants are provided resources to safely explore their environment, develop problem-solving, practice self-agency, and retreat to caregivers for safety in times of distress, this contributes to a secure attachment pattern [8,16]. Insecure attachment develops when an infant’s needs are not adequately met, including through insufficient attention, rejection, abuse, or inconsistent caregiving [8,16]. These patterns largely persist into adulthood, but attachment security or insecurity may change due to experiences over the life course, such as positive intimate relationships or the onset of life-threatening illness [7]. In addition, epigenetics may play a role in shaping these patterns, and importantly, these patterns are also influenced by broader social, cultural, economic, and political factors [17,18].

**Figure 1.**
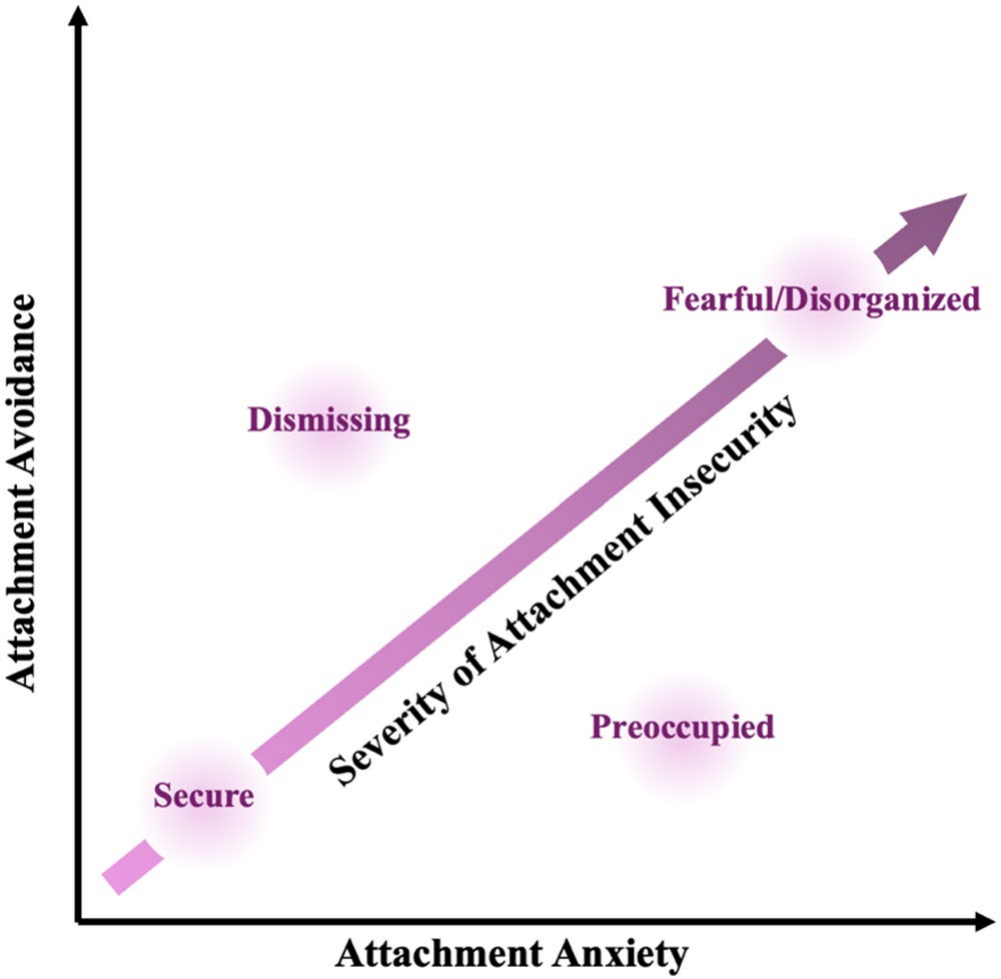
Prototype-based model of adult attachment for clinicians. Adapted from Maunder and Hunter [16].

In adulthood, a secure attachment style is marked by a positive sense of self and others with balanced levels of independence, seeking support when needed, and comfort with closeness. Insecure adult attachment can be marked by anxiety (a negative sense of self: clinginess, preoccupation with relationships) and/or avoidance (a negative sense of others: excessive independence, devaluing of close relationships) [14,16]. The prototype-based model of adult attachment provides a guide for assessing and considering attachment styles in clinical contexts [16,19]. Central to the model are the two dimensions of attachment insecurity – attachment anxiety and attachment avoidance – which reflect underlying differences in internal working models of the self and others, as well as affect regulation, behavior in close relationships, narrative coherence, and broader psychological capacities such as mentalizing and self-agency. Four prototypic attachment patterns – secure, fearful/disorganized, dismissing, and preoccupied – are considered in this conceptual space (Figure 1) [14,16].

The prototype-based approach accommodates both categorical and dimensional understandings of attachment while emphasizing the clinical relevance of the degree of attachment insecurity [16]. Measures for assessing adult attachment have been detailed elsewhere [20]. Briefly, self-report questionnaires have been shown to have good psychometric properties. In care contexts, clinicians familiar with attachment theory may identify attachment styles by the main characteristics of the prototypes without necessarily needing formal measurement tools [16,19].

In the face of threats to perceived safety, like severe illness, attachment theory becomes even more relevant. In CKD, illness-related fear, vulnerability, and isolation may activate the attachment system and often trigger attachment behaviors. A growing body of evidence links attachment insecurity with the onset of chronic disease and poorer self-management and outcomes [7,9,11]. However, very few studies have investigated adult attachment in the context of CKD. Here, we discuss published data and highlight the need for research in this patient population that considers the role of adult attachment, highlighting CKD onset and management as two foci of inquiry.

## Adult attachment patterns and CKD onset

Maunder and Hunter [8] highlight three pathways through which insecure attachment can contribute to disease: (i) greater susceptibility to stress, including the perception of stress and more extreme cognitive responses to stress; (ii) tendency to use external affect regulators, namely substance use, eating behaviors, and risky sexual behaviors, which can cause health concerns and (iii) poorer protective health behaviors, including underutilization of social supports and health care services, poorer treatment adherence and less symptom reporting [8]. There is significant and growing empirical evidence in support of these pathways [7,9,11].

Thirty-year longitudinal evidence links insecure attachment with physical illnesses in general, and specifically with inflammation-related disease in adulthood after controlling for gender, socioeconomic status, body mass index, social support, life stress, and negative emotional style [21]. Similar longitudinal research highlights the potential association of attachment insecurity in early adulthood with cardiometabolic risk factors at midlife [22]. Cross-sectional evidence from a nationally representative sample of non-institutionalized US adults demonstrated the association between anxious attachment and cardiovascular conditions (stroke, heart attack, and hypertension) after controlling for depression, anxiety, and substance use [23].

Importantly, not only has attachment insecurity been linked to developing the above risk factors for CKD, but also among patients with diabetes, attachment insecurity is associated with higher distress, more daily stressors, lower self-care, increased smoking, worse adherence to treatment recommendations, and worse glucose control [7,24]. Additionally, in a prospective study of older adults (aged 50–85) with multimorbidity (type II diabetes, hypertension, and at least one other chronic disease), anxious attachment was negatively associated with coping, self-efficacy, hope, dietary control, and physical activity. Attachment avoidance was negatively associated with coping and self-efficacy, as well as social support and health care use [25].

Attachment insecurity is associated with the development and poorer management of CKD risk factors. This may potentially contribute to the development of CKD. To our knowledge, no prior studies have directly examined attachment insecurity as a predictor of CKD onset. Longitudinal studies with representative samples are needed to investigate attachment insecurity as a potential contributing factor in CKD onset and early progression. Social support, mental health concerns, clinical factors, genetic risks, and other psychosocial and biological factors are potential contributing variables that should be considered in future studies.

## Adult attachment patterns and the management of CKD

Attachment insecurity may have an impact on the management and progression of CKD. Among patients with advanced CKD on maintenance dialysis, insecure attachment is associated with greater levels of psychosocial distress, lower HRQoL and non-adherence to treatment recommendations [26–30]. Secure attachment is associated with higher mental HRQoL [27]. Attachment insecurity and its effects may also impact access to KT as well as post-transplant outcomes. Among patients with advanced CKD considering KT, secure attachment is associated with a lower predicted risk of post-transplant psychosocial challenges [31]. Small studies of KT recipients suggest that insecure attachment is correlated with lower HRQoL [32,33], and that attachment anxiety moderates the relationship between coping strategies and illness acceptance in this population [34]. Further, preliminary research with 43 KT recipients indicates that insecure attachment may be associated with poorer adherence to immunosuppressant regimens and anxious attachment specifically may be associated with poorer kidney function; however, this finding requires further investigation [33].

The relationship between attachment insecurity and other post-transplant outcomes including anxiety regarding graft loss and broader clinical outcomes remains limited and warrants further investigation. In addition, considering that attachment patterns are further developed through one’s life course and impact one’s ability to respond to stressors, continued experiences of adversity, such as poverty and experiencing racism, may worsen the negative impact of insecure attachment in some patients with CKD, potentially contributing to poorer health outcomes.

Increasingly, research highlights that for patients with chronic disease, good patient–clinician relationships may ameliorate the association between insecure attachment and poorer health outcomes [7,29]. Knowledge of attachment prototypes and related psychopathology may equip clinicians with skills to develop better rapport with patients to achieve mutually desired health outcomes [7,16]. In addition, attachment theory may help clinicians better identify patients at increased risk for severe distress and provide referrals for psychosocial interventions as needed [7]. In the context of psychosocial interventions, an attachment lens may prove useful in optimizing treatments for patients with insecure attachment. Evidence-based approaches that focus on stress regulation and reactivity, for example, may be particularly helpful [9].

Published results from cross-sectional studies suggest that attachment patterns may have significant implications for the health of patients with advanced CKD. Living with CKD and undergoing treatments for this condition, namely dialysis and KT, is stressful and may activate the attachment system, contributing to worse stress responses. Adult attachment may therefore be relevant along the kidney care continuum. Attachment theory may inform tailoring interventions to slow disease progression, comfort-focused management of advanced CKD, as well as to prepare patients for KT or dialysis. Considering adult attachment patterns during the pre-transplant assessment may better inform our understanding of how a patient will cope, adhere to their treatment and manage the mental and physical symptoms present after transplantation [31]. Furthermore, there is need for empirical study of adult attachment in patients with early-stage CKD to better understand how attachment patterns may influence disease trajectory.

Attachment patterns may also influence treatment decisions by patients, specifically live donor KT (LDKT). Patients with secure attachment are possibly more likely to consider LDKT, since they may feel more comfortable communicating their need for a live donor and accepting a potential donor offer. This, however, will need to be tested in future empirical research. Additionally, attachment patterns may also influence an individual’s willingness to donate an organ. To our knowledge, no studies have directly examined the relationship between attachment patterns and either living kidney donation or registration for deceased donation. Given that attachment patterns shape internal working models of self and others and therefore interpersonal behavior, this may be a relevant framework for understanding decision-making in this context. This represents an important and interesting area for future research.

## Conclusions

More research is needed about attachment patterns in patients with CKD. Published longitudinal and cross-sectional evidence indicates a link between insecure attachment and CKD risk factors and poorer disease management. Cross-sectional evidence has linked attachment insecurity with poorer outcomes among patients with CKD. Further studies, particularly longitudinal studies, are needed to better understand the role attachment patterns may play in kidney health and to inform appropriate interventions. Consideration of attachment patterns in kidney care may help to improve whole-person, patient-centered approaches to both prevent CKD and better support patients living with CKD.

## Data Availability

No data were generated in the production of this article.

## References

[1] GBD 2023 Chronic Kidney Disease Collaborators. Global, regional, and national burden of chronic kidney disease in adults, 1990–2023, and its attributable risk factors: a systematic analysis for the Global Burden of Disease Study 2023. Lancet. 2025;406(10518):2461–2482. 41213283 10.1016/S0140-6736(25)01853-7

[2] Okpechi IG, Levin A, Tungsanga S, et al. Progress of nations in the organisation of, and structures for, kidney care delivery between 2019 and 2023: cross sectional survey in 148 countries. BMJ. 2024;387:e079937. doi:10.1136/bmj-2024-079937. 39401841 PMC11472216

[3] Fletcher BR, Damery S, Aiyegbusi OL, et al. Symptom burden and health-related quality of life in chronic kidney disease: a global systematic review and meta-analysis. PLoS Med. 2022;19(4):e1003954. doi:10.1371/journal.pmed.1003954. 35385471 PMC8985967

[4] de Jong Y, van der Willik EM, Milders J, et al. Person centred care provision and care planning in chronic kidney disease: which outcomes matter? A systematic review and thematic synthesis of qualitative studies: care planning in CKD: which outcomes matter? BMC Nephrol. 2021;22(1):309. doi:10.1186/s12882-021-02489-6. 34517825 PMC8438879

[5] Gill JK, Pucci M, Samudio A, et al. Self-reported MeasUrement of Physical and PsychosOcial Symptoms Response Tool (SUPPORT-dialysis): systematic symptom assessment and management in patients on in-centre haemodialysis – a parallel arm, non-randomised feasibility pilot study protocol. BMJ Open. 2024;14(1):e080712. doi:10.1136/bmjopen-2023-080712. PMC10828879 38296283

[6] Tang E, Yantsis A, Ho M, et al. Patient-reported outcome measures for patients with CKD: the case for patient-reported outcomes measurement information system (PROMIS) tools. Am J Kidney Dis. 2024;83(4):508–518. doi:10.1053/j.ajkd.2023.09.007. 37924931

[7] Jimenez XF. Attachment in medical care: a review of the interpersonal model in chronic disease management. Chronic Illn. 2017;13(1):14–27. doi:10.1177/1742395316653454. 27269506

[8] Maunder RG, Hunter JJ. Attachment and psychosomatic medicine: developmental contributions to stress and disease. Psychosom Med. 2001;63(4):556–567. doi:10.1097/00006842-200107000-00006. 11485109

[9] Meredith PJ, Strong J. Attachment and chronic illness. Curr Opin Psychol. 2019;25:132–138. doi:10.1016/j.copsyc.2018.04.018. 29753973

[10] Pietromonaco PR, Uchino B, Dunkel Schetter C. Close relationship processes and health: implications of attachment theory for health and disease. Health Psychol. 2013;32(5):499–513. doi:10.1037/a0029349. 23646833 PMC3648864

[11] Maunder RG, Hunter JJ. Attachment relationships as determinants of physical health. Psychodyn Psychiatry. 2022;50(2):360–379. doi:10.1521/pdps.2022.50.2.360. 35653525

[12] Bowlby J. Attachment and loss. Vol. 1. Attachment. New York: Basic Books; 1969.

[13] Ainsworth MDS, Blehar MC, Waters E, et al. Patterns of attachment: a psychological study of the strange situation. Hillsdale (NJ): Erlbaum; 1978.

[14] Bartholomew K, Horowitz LM. Attachment styles among young adults: a test of a four-category model. J Pers Soc Psychol. 1991;61(2):226–244. doi:10.1037/0022-3514.61.2.226. 1920064

[15] Ainsworth MDS, Bowlby J. An ethological approach to personality development. Am Psychol. 1991;46(4):333–341. doi:10.1037/0003-066X.46.4.333.

[16] Maunder RG, Hunter JJ. A prototype-based model of adult attachment for clinicians. Psychodyn Psychiatry. 2012;40(4):549–573. doi:10.1521/pdps.2012.40.4.549. 23216396

[17] Erkoreka L, Zumarraga M, Arrue A, et al. Genetics of adult attachment: an updated review of the literature. World J Psychiatry. 2021;11(9):530–542. doi:10.5498/wjp.v11.i9.530. 34631458 PMC8474999

[18] Stern JA, Barbarin O, Cassidy J. Working toward anti-racist perspectives in attachment theory, research, and practice. Attach Hum Dev. 2022;24(3):392–422. doi:10.1080/14616734.2021.1976933. 34528474 PMC8924009

[19] Maunder RG, Hunter JJ. Assessing patterns of adult attachment in medical patients. Gen Hosp Psychiatry. 2009;31(2):123–130. doi:10.1016/j.genhosppsych.2008.10.007. 19269532

[20] Ravitz P, Maunder R, Hunter J, et al. Adult attachment measures: a 25-year review. J Psychosom Res. 2010;69(4):419–432. doi:10.1016/j.jpsychores.2009.08.006. 20846544

[21] Puig J, Englund MM, Simpson JA, et al. Predicting adult physical illness from infant attachment: a prospective longitudinal study. Health Psychol. 2013;32(4):409–417. doi:10.1037/a0028889. 22823067 PMC3480992

[22] Farrell AK, Waters TEA, Young ES, et al. Early maternal sensitivity, attachment security in young adulthood, and cardiometabolic risk at midlife. Attach Hum Dev. 2019;21(1):70–86. doi:10.1080/14616734.2018.1541517. 30428778 PMC13091069

[23] McWilliams LA, Bailey SJ. Associations between adult attachment ratings and health conditions: evidence from the National Comorbidity Survey Replication. Health Psychol. 2010;29(4):446–453. doi:10.1037/a0020061. 20658833

[24] Kelly CS, Berg CA, Helgeson VS. Adult attachment insecurity and associations with diabetes distress, daily stressful events and self-management in type 1 diabetes. J Behav Med. 2020;43(5):695–706. doi:10.1007/s10865-019-00111-7. 31641989 PMC7174097

[25] Brenk-Franz K, Strauss B, Tiesler F, et al. The influence of adult attachment on patient self-management in primary care – the need for a personalized approach and patient-centred care. PLOS One. 2015;10(9):e0136723. doi:10.1371/journal.pone.0136723. 26381140 PMC4575213

[26] Novak M, Ford H, Edwards N, et al. Adult attachment styles and psychosocial distress in patient with end stage kidney disease (ESKD). Psychosom Med. 2019;121:132–133. doi:10.1016/j.jpsychores.2019.03.099.

[27] De Pasquale C, Pistorio ML, Veroux M, et al. Attachment and parental bond: impact on psychopathology, mental health and quality of life of hemodialysis patients: a cross-sectional study. BMC Psychol. 2023;11(1):210. doi:10.1186/s40359-023-01246-8. 37454118 PMC10349506

[28] Swaby DJ, El-Sururi M, Syeda N, et al. The impact of adult attachment insecurity on health related quality of life (HRQoL) in patients with advanced chronic kidney disease (CKD): a cross-sectional analysis. J Psychosom Res. 2025;196:112282. doi:10.1016/j.jpsychores.2025.112282.

[29] Fuertes JN, Rubinstein S, Reyes M, et al. The physician–patient working alliance in hemodialysis treatment. Behav Med. 2017;43(4):242–250. doi:10.1080/08964289.2015.1122569. 26808407

[30] Alosaimi FD, Asiri M, Alsuwayt S, et al. Psychosocial predictors of nonadherence to medical management among patients on maintenance dialysis. Int J Nephrol Renovasc Dis. 2016;9:263–272. doi:10.2147/IJNRD.S121548. 27826207 PMC5096770

[31] Pistorio ML, De Pasquale C, Veroux M, et al. The role of attachment and parental bonding in the psychosocial assessment of transplant candidates: a cross-sectional study. BMC Psychol. 2025;13(1):227. doi:10.1186/s40359-025-02558-7. 40069790 PMC11895345

[32] Calia R, Lai C, Aceto P, et al. Emotional management and quality of life in mother living versus multi-organ donor renal transplant recipients. J Health Psychol. 2017;22(4):475–482. doi:10.1177/1359105315604378. 26430068

[33] Calia R, Lai C, Aceto P, et al. Attachment style predict compliance, quality of life and renal function in adult patients after kidney transplant: preliminary results. Ren Fail. 2015;37(4):678–680. doi:10.3109/0886022X.2015.1010989. 25687387

[34] Hamama-Raz Y, Bergman YS, Ben-Ezra M, et al. Attachment patterns moderate the relation between coping flexibility and illness acceptance among kidney transplant recipients. Anxiety Stress Coping. 2018;31(5):571–579. doi:10.1080/10615806.2018.1498667. 30012024

